# Exploiting Fruit Waste Grape Pomace for Silver Nanoparticles Synthesis, Assessing Their Antioxidant, Antidiabetic Potential and Antibacterial Activity Against Human Pathogens: A Novel Approach

**DOI:** 10.3390/nano10081457

**Published:** 2020-07-25

**Authors:** Ganesh Dattatraya Saratale, Rijuta Ganesh Saratale, Dong-Su Kim, Do-Yeong Kim, Han-Seung Shin

**Affiliations:** 1 Department of Food Science and Biotechnology, Dongguk University, Ilsandong-gu, Goyang-si, Gyeonggido, Seoul 10326, Korea; gdsaratale@dongguk.edu; 2 Research Institute of Biotechnology and Medical Converged Science, Dongguk University, Ilsandong-gu, Goyang-si, Gyeonggido, Seoul 10326, Korea; rijutaganesh@gmail.com; 3 Department of Environmental Science and Engineering, Ewha Womans University, Seoul 120-750, Korea; dongsu@ewha.ac.kr

**Keywords:** grape pomace, silver nanoparticles, cytoplasmic leakages, antidiabetic activity, antioxidant potential

## Abstract

Grape pomace, a most abundant and renewable wine industry waste product was utilized as a suitable reducing, capping, and stabilizing biomolecules for green synthesis of silver nanoparticles (AgNPs). The physicochemical properties of biosynthesized grape pomace extract (GPE)-AgNPs were duly appraised via UV–Visible spectroscopy, X-ray diffractometer (XRD), Fourier-transform infrared spectroscopy (FTIR), and transmission electron microscopy. The analytical studies revealed that the GPE-AgNPs were well formed and stable in nature. The functional groups of organic molecules of GPE are present on the surface of AgNPs with average NPs diameter in the range of 20–35 nm. GPE-AgNPs exhibited significant free radical scavenging activity mainly DPPH radical (IC_50_, 50.0 ± 2.25 μg/mL) and ABTS radical (IC_50_, 38.46 ± 1.14 μg/mL). Additionally, the synthesized AgNPs showed noticeable inhibition of carbohydrate hydrolyzing enzymes mainly, α-amylase (IC_50_, 60.2 ± 2.15 μg/mL) and α-glucosidase (IC_50_, 62.5 ± 2.75 μg/mL). The GPE fabricated AgNPs showed noteworthy antibacterial potential against infectious bacteria viz., *Escherichia coli* and *Staphylococcus aureus*. The reaction mechanism of antibacterial activity was studied by measuring the bacterial cell membrane breakage and cytoplasmic contents, mainly, nucleic acid, proteins, and reducing sugar. Therefore, this research attempt illustrated the potential of GPE as a novel source intended for the biosynthesis of AgNPs that may open up new horizons in the field of nanomedicine.

## 1. Introduction

The synthesis of nanoscale materials with different structural configurations and unique physicochemical attributes are receiving escalating recognition. This may be because of its versatile applications in numerous prominent areas such as life sciences, material sciences, biomedical engineering, wastewater treatment, biosensors development, chemistry, and physics [1]. Silver nanoparticles (AgNPs) are well recognized metal nanoparticles (NPs) because of its exceptional biocompatibility and have demonstrated numerous properties such as antimicrobial, wound healing, biosensors, drug delivery agents, in biomedical devices, and as a catalyst [2,3,4,5]. Conversely, application of AgNPs in various sectors leads to environmental pollution and showed toxicity to the ecosystem and human health [6]. NPs synthesis and their fabrication can be performed through numerous physicochemical methods, which are usually not economical and end up with lethal by-products [7]. Green nanotechnology following green chemistry principles are gaining significant momentum because of certain advantages including easy, faster, clean, efficient, and sustainable process and provides low adverse effects compared to the synthetic sources [8]. Several routes of green synthesis of AgNPs by employing various biological materials such as microorganisms, whole plants, extracts of different parts of plants, fruit waste, and marine algae have been well investigated. Among which, utilization of plant- or fruit waste-based extracts could be more beneficial due to simple, cost-effective, and eco-accommodating process relative to other biological routes of NPs synthesis [8,9]. There are a few reports of green synthesis of silver nanoparticles using leaf extract of *Holoptelea integrifolia*, *Punica granatum*, waste fruit extract of *Vitis vinifera*, and using single biomolecules such as gallic acid, chlorotetracycline, and lignin [1,2,3,4,5,8,9,10,11,12,13]. The synthesized AgNPs have been studied for various biogenic activities which increases its potential applications in biomedical, environment sector.

Grape (*Vitis vinifera*) is among the world’s major fruits and is extensively utilized in juice and wine industries. During processing, a significant amount of solid by-product, namely, grape pomace (GP) is generated [14,15]. However, recycling of GP is still limited and is mainly utilized as cattle feed, for composting, or throw out in open areas, which consequently leads to environment pollution [14,16]. The GP consists of grape skins and seeds those are rich in phytochemicals, namely, phenolic acids, flavonoids, anthocyanins, resveratrol, and proanthocyanidins [17,18]. The presence of these valuable phytochemicals makes GP as a vital source for the green synthesis of AgNPs.

Developments of multidrug-resistant bacterial strains primarily caused by mutation and environmental pollution led to major threats to human health. AgNPs exhibited excellent antibactericidal potential to curb such a situation because of its large surface area, high fraction of surface atoms with low toxicity in human beings [19]. In human body, oxidation is essential to maintain biological processes for the production of energy. During cellular metabolic processes, reactive oxygen species (ROS), mainly, superoxide radical (O_2_^•−^), hydroxyl radical (^•^OH), and hydrogen peroxide H_2_O_2_, are produced. ROS at lower level are beneficial for cellular growth, metabolism, signaling, and development, however, imbalance among production and accumulation resulted in oxidative stress [20]. Excess free radicals generation is accountable for cell death, tissue damage, and many degenerative diseases. Antioxidants are proficient of deactivating free radicals and intriguing in defensive function in cell injury endorsed by free radicals-induced oxidative stress. In diabetic patients, elevated oxidative stress and hyperglycemia leads to excess accumulation of ROS, which causes the cellular injury, tissue damage, or organ dysfunctions [21]. ROS are liable for insulin resistance, β-cell dysfunction, and also for both microvascular and macrovascular long-term complications of diabetes [22]. Green synthesized AgNPs fabricated with phytochemicals showed significant in vitro antioxidant potential, but yet their medical importance has not been scrutinized well [9,23].

Based on the above stated facts, the present work was intended to develop and validate simple, economical, eco-friendly fabrication of AgNPs using fruit waste material grape pomace extract (GPE). The physicochemical properties of synthesized GPE-AgNPs were characterized using various spectroscopic and microscopic analytical methods. In addition, GPE-AgNPs were assessed for the antibacterial potential against human pathogenic bacteria and also investigated for their underlying molecular mechanism. Further, the biosynthesized AgNPs were explored for the antioxidant and antidiabetic activity to raise their biomedical preponderance. This research work would give useful recognition on utilizing GP as a valuable resource for AgNPs synthesis and their possible applications in the medical sector.

## 2. Materials and Methods

### 2.1. Chemicals and Microbial Strains

Silver nitrate (AgNO_3_), ascorbic acid, 2,2-diphenyl-1-picryhydrazyl (DPPH), 2,2′-Azino-bis(3-ethylbenzothiazoline)-6 sulfonic acid (ABTS), sodium potassium tartrate, 3,5-dinitrosalicylic acid (DNS), gallic acid, querecitin, acarbose, α-glucosidase, α-amylase, and Folin–Ciocalteu’s phenol reagent (2 N) were purchased from Sigma-Aldrich, St. Louis, MO, USA. All other chemicals requisite for all experimentation were of analytical grade and high purity. The purified water was utilized for the entire experiment (Millipore Corporate, Billerica, MA, USA). Human pathogenic strains, namely, *Staphylococcus aureus* and *Escherichia coli* were used for the determination of antibacterial potential of biosynthesized AgNPs.

### 2.2. Collection and Processing of Grape Pomace

Grape pomace (GP) was collected from the local wine industry (Sangli, India). The obtained GP was dried in an oven at 550 °C (since this temperature may not influence the phytochemicals present in the GP) until the constant weight achieved. The dried GP was pulverized into fine small pieces and stored in sealed plastic bags at 40 °C until further use. For the organic solvent extraction of phytochemicals, the GP powder was immersed in pure ethanol in 1:20 proportion. The reaction mixture was placed under constant stirring (200 rpm) for 24 h under dark condition. Afterwards, the reaction suspension was centrifuged at 5000× *g* for 15 min, and the supernatant was concentrated in the rotary evaporator. Finally, samples were lyophilized and a violet-brown hygroscopic powder was stored in dark for subsequent procedures.

### 2.3. Estimation of Total Phenolic Content of GPE

The Folin–Ciocalteu procedure was applied, due to its high sensitivity and simplicity, to assess the total phenolic content (TPC) of GPE [24]. Briefly, in microtiter plate the reaction mixture consists of 20 µL of GPE and 180 μL distilled water was added to 100 µL of Folin-Ciocalteu reagent and 200 μL of sodium carbonate (20% w/v). Further, the reaction suspension was incubated at room temperature and the absorbance was measured at 765 nm against reagent blank containing water and gallic acid as a standard. A calibration curve with various concentrations of gallic acid (20–100 μg/mL) (absorbance vs. μg/mL) was used for TPC content measurement. The TPC concentration was presented in mg of gallic acid equivalent (GAE)/g of GPE. 

### 2.4. Estimation of Total Flavonoids Content of GPE

For the determination of total flavonoids content (TFC) present in GPE, aluminum chloride colorimetric manner method illustrated by [25] was employed. An aliquot of 120 μL of GPE was added to 120 μL of aluminum chloride (2%, w/v) in a microtiter plate, and absorbance of the reaction solution was deliberated at 415 nm using a microplate reader after 10 min of incubation. Quercetin was employed as a standard to estimate the TFC present in the GPE and was conferred in mg of quercetin equivalent (QE)/g of GPE.

### 2.5. GPE-AgNPs Synthesis

Silver nitrate (AgNO_3_, 1 mM) solution and aqueous GPE extract were mixed together in a ratio of 20:1 in a 500 mL of Erlenmeyer conical flask. The reaction suspension was mixed well and incubated at 500 °C for 20 min for the biofabrication of AgNPs. The succession of GPE-AgNPs synthesis was established indicating a change in color from light yellow to dark brown in 20 min. The colored reaction mixture obtained after the process was centrifuged for 20 min at 12,000 rpm to remove the unreacted reductants (Labogene, 1736R, Lillerød, Denmark). The AgNPs was washed twice with Milli-Q water and the obtained pellet of the AgNPs was dried in an oven at 600 °C and further stored in sealed vial at 4 °C until further use.

### 2.6. Characterization Studies

The surface plasmon resonance property of the GPE-AgNPs was confirmed by recording the absorbance spectra in the range of 300–650 nm at fixed time intervals by UV–Visible spectrophotometer, Optizen, Model-2120. Crystallinity and phase of the AgNPs were characterized by X-ray diffractometer (Rigaku, Tokyo, Japan) [9]. Fourier-transform infrared (FTIR) spectra of the GPE and synthesized GPE-AgNPs were recorded by taking spectra in the range of 400–4000 cm^−1^ on Fourier-transform infrared spectrophotometer (Perkin-Elmer, Norwalk, CT, USA) to identify the organic functional groups present on synthesized AgNPs. The size, imaging, and exterior arrangement of synthesized GPE-AgNPs was interpreted by high-resolution transmission electron microscopy (HRTEM, Tecnai G2 20 S-TWIN, FEI Company, Loughborough, UK). The particle size and shape of GPE-AgNPs was evaluated by measuring the median size of about 100 diverse NPs acquired in TEM image [26].

### 2.7. In Vitro Antioxidant Assays

Antioxidative potential of GPE, and synthesized GPE-AgNPs was evaluated by assessing their potential to scavenge the stable free radicals like 2,2-diphenyl-1-picrylhydrazyl (DPPH) and 2,2-Azino-bis(3-ethylbenzothiazoline)-6 sulfonic acid (ABTS), taking standard as ascorbic acid. The procedure of both the enzyme assays and the DPPH and ABTS scavenging potential were determined using the formulas reported earlier [9]. For both enzyme activities, average mean values and standard deviation values were measured to calculate the antioxidant activity of GPE-AgNPs.

### 2.8. In Vitro Antidiabetic Activity

The green synthesized GPE-AgNPs was evaluated for antidiabetic potential by determining α-amylase and α-glucosidase enzyme inhibitory activity. The α-amylase activity was measured by standard DNS method [27]. In brief, an aliquot of 500 μL of different concentrations of GPE-AgNPs (20, 40, 60, 80, and 100 µg/mL) were mixed in 500 μL of α-amylase (1 U/mL) solution and 500 μL of 0.02 M Na_3_PO_4_ buffer (pH 6.9) followed by addition of 500 μL of starch solution, and the reaction mixture was further incubated for 20 min at 37 °C. Dinitrosalicylic acid color reagent (1 mL) was added to the above test to terminate the reaction and heated in boiled water for 10 min. It was further incubated at room temperature to cool down and finally, the absorbance was measured at 540 nm.

Biogenic AgNPs were also investigated for its ability to inhibit the α-glucosidase enzyme activity. The study depends on the hydrolysis of PNPG by α-glucosidase to yellow-colored p-nitrophenyl [28]. Solutions of α-glucosidase (1 unit/mL) and PNPG (5 mM) were reacted in phosphate buffer (100 mM, pH 6.9). Then 100 μL of the α-glucosidase was added with 50 μL of different concentrations (20, 40, 60, 80, and 100 µg/mL) of GPE-AgNPs, 150 μL Na_3_PO_4_ buffer (0.1 M; pH 6.9), and 50 μL of PNPG (2 mM) and incubated at 37 °C for 20 min. The absorbance was noted at 405 nm, and acarbose was taken as standard for both enzyme activities. The average mean ± standard error mean values were documented to calculate the antidiabetic activity of GPE-AgNPs.

### 2.9. In Vitro Antibacterial Activity

In vitro investigations for antibacterial potency of biofabricated NPs contrary to Gram-negative (*Escherichia coli*) and Gram-positive (*Staphylococcus aureus*) bacteria was carried out by following the typical Kirby-Bauer disc diffusion procedure [29]. The used bacterial species were cultivated on nutrient agar slants and stored at 4 °C in the laboratory. The antibacterial activity of GPE-AgNPs was performed by determining the zone of inhibition (ZOI). Sterile Whatman-1 filter paper discs (5 mm diameter) were used to load NPs solution (10, 15, and 20 μL) from stock solution (2 mg/mL). Precisely, 100 μL of freshly grown overnight cultures were spread over sterile nutrient agar plates. The plates were allowed to stand for 1 h for the perfusion of the samples and were kept at 37 °C for 24 h for incubation. The ZOI, in millimeter, surrounding the discs were measured. The antibacterial potency of the biofabricated NPs was proportional to their ZOI. Deionized water was considered as negative control and GPE as positive control. Antibacterial potency of NPs was also investigated in combination with the standard reference antibiotics such as ampicillin, tetracycline, kanamycin, vancomycin, and streptomycin by applying filter paper discs loaded with both NPs (20 μg/mL) and antibiotics (5 μL of each) from freshly prepared stock solution (2 mg/mL). Discs only with antibiotics (positive) and NPs (negative) were also used as controls. Synergistic activity was quantified with percentage (%) fold increase in ZOI [30].

### 2.10. Molecular Mechanisms of GPE-AgNPs-Mediated Antibacterial Activity

#### 2.10.1. Disruption of Bacterial Membrane and Cytoplasmic Leakages

The basics of antibacterial activity by GPE-AgNPs was studied spectrophotometrically by measuring bacterial cell membrane integrity and the leakage of cellular components such as nucleic acids (RNA/DNA), cellular proteins, and reducing sugar after 6 h of incubation. Absorbance of each pathogenic bacterial culture (freshly grown overnight) was adjusted to ~1.0 at 600 nm and the bacterial cells were collected from 10 mL by centrifugation. The cells were subsequently preserved with different concentrations of GPE-AgNPs (25–100 μg/mL) and incubated at 37 °C for 6 h. After GPE-AgNPs exposure, the samples were centrifuged (10 min, 5000 rpm) to get rid of bacterial cells and NPs. Bacterial cell membrane integrity and release of nucleic acid material was examined by recording the absorbance at 260 and 280 nm [31]. Resultant supernatant was diluted (20×) before measuring the absorbance. Sterile water was used as a control. Further, the release of reducing sugar and proteins concentration of the resultant bacterial supernatant were determined by using DNS procedure and Bradford assay, respectively [32,33].

#### 2.10.2. Statistical Analysis

All the experiments were implemented in triplicate and the results are conferred as mean ± standard error mean (SEM) of all calculated values. The data obtained were interpreted by employing the one-way analysis of variance (ANOVA) test accompanied by a Tukey–Kramer multiple comparisons test.

## 3. Results and Discussion

### 3.1. Determination of Phytochemical Constituents in GPE

The phytochemical constituents and its concentration in grape pomace depend on the grape varieties and the extraction procedure. In the present study, organic solvent extraction procedure was applied to extract phytochemicals from GP. Further, the main phytochemical biomolecules such as TPC and TFC in GPE were determined. The results of chemical constituents of GPE are presented in Table 1.

The results proved that GPE contain high amount of phenolic compounds and moderate concentration of total flavonoids. The obtained value of TPC and TFC are consistent with the results obtained with other studies [34,35]. TPC and TFC are natural antioxidants and are therapeutically very useful biomolecules due to their redox properties with many health-beneficial effects. The analysis also demonstrated that TPC and TFC of GPE acts as reducing and capping agents in the biofabrication process and also prevents the agglomeration of the synthesized GPE-AgNPs.

### 3.2. Synthesis and Characterization of GPE-AgNPs

**Figure 1:**
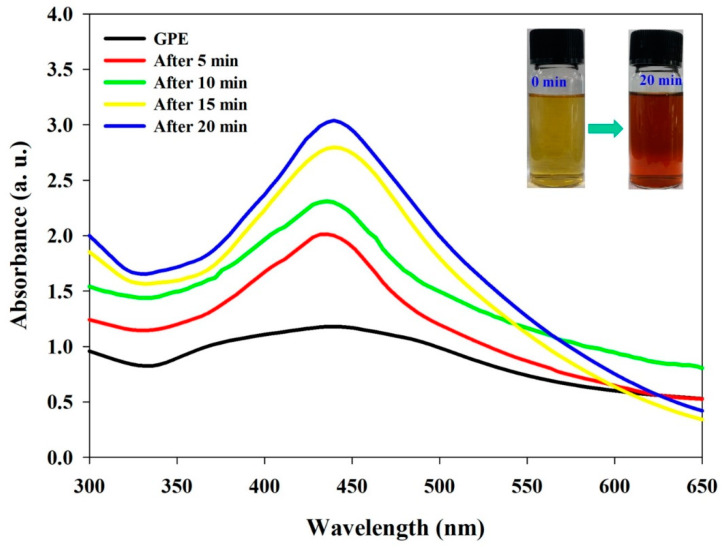
UV–visible absorption spectrum of synthesized grape pomace extract-silver nanoparticles (GPE-AgNPs) at different time intervals.

The present work was conducted to biofabricate the AgNPs in a single step employing grape pomace solvent extract (GPE) as a reducing and capping agent. The UV–visible absorption spectroscopy is a simple and sensitive method to examine the formation of NPs. After addition of GPE in silver nitrate solution, development in color (pale yellow to dark brown) of the reaction mixture (Figure 1 inset) indicates the formation of GPE-AgNPs. The progression in color change of the mixture is because of surface plasmon resonance (SPR) and vibration of the synthesized AgNPs. The absorption spectra during GPE-AgNPs synthesis at different time intervals are showed in Figure 1, wherein, the SPR showed a distinct peak at 440 nm. According to Mie’s theory, the number of SPR relies on the shape of NPs and a single peak indicates the spherical GPE-AgNPs formation [36]. This result was also found similar with silver nanoparticles synthesized using *Lawsonia inermis* leaf extract [37].

**Figure 2:**
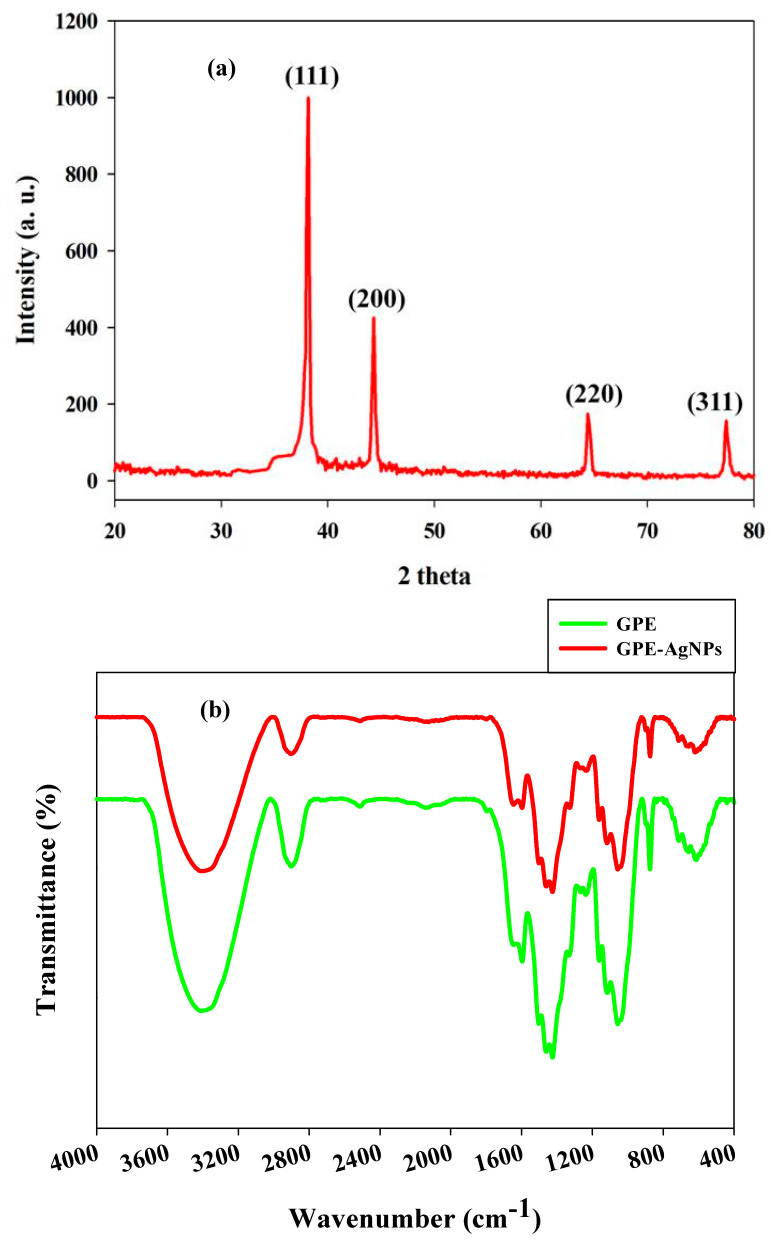
(**a**) X-ray diffraction pattern of synthesized GPE-AgNPs and (**b**) FTIR spectrum of GPE and synthesized GPE-AgNPs.

The synthesized GPE-AgNPs crystalline nature was confirmed by XRD analysis. Figure 2a depicted X-ray diffraction pattern of the synthesized GPE-AgNPs. Four Bragg’s reflections with 2θ values of 38.3°, 44.6°, 64.6°, and 77.6° were observed, which were readily be indexed to the planes (111), (200), (220), and (311), respectively. The results also confirmed that the synthesized GPE-AgNPs were of crystalline face-centered cubic (FCC) nature according to the catalog of the Joint Committee on Powder Diffraction Standards (JCPDS file No. PDF#030921). Absence of any other peak easily validated phase purity of the synthesized GPE-AgNPs. The outcomes of the results were in agreement with the results revealed by other reports [26,37].

FTIR analysis was performed to identify the function of biomolecules of grape pomace in GPE-AgNPs synthesis. The functional groups liable for the bioreduction and capping of the biogenic AgNPs, which leads to efficient stabilization were investigated. FTIR spectra of GPE and GPE-AgNPs are depicted in Figure 2b. Broad absorption bands of hydroxyl groups were found around 3354 cm^−1^ attributed to O-H group in polyphenols or polysaccharide for both GPE and synthesized GPE-AgNPs [15,38]. A small peak deliberated at 2922 cm^−1^ is related to the CH-stretching of alkanes [15]. The band at 1610 cm^−1^ was assigned to stretching vibration of C-OH bond from proteins or aromatic squeal vibration [16]. A peak at 1417 cm^−1^ correlated to C-N stretching of aromatic amine rings, whereas vibration band at 1059 cm^−1^ was assigned to the hydroxyl group in carbohydrate [39]. The intense peak of 628 cm^−1^ represents the C-Cl group of alkyl halides, and a band at 878 cm^−1^ exhibited for aromatic ring vibration [40]. The FTIR analysis supports the presence of polyphenols, protein, and saccharides of GPE, which play a vital role in fabricating and stabilizing agent in the formation of GPE-AgNPs. The results also suggest that GPE biomolecules have stronger ability to bind with silver, leading to form a layer covering the synthesized AgNPs and to prevent the agglomeration of NPs.

### 3.3. High-Resolution Transmission Electron Microscopy

**Figure 3:**
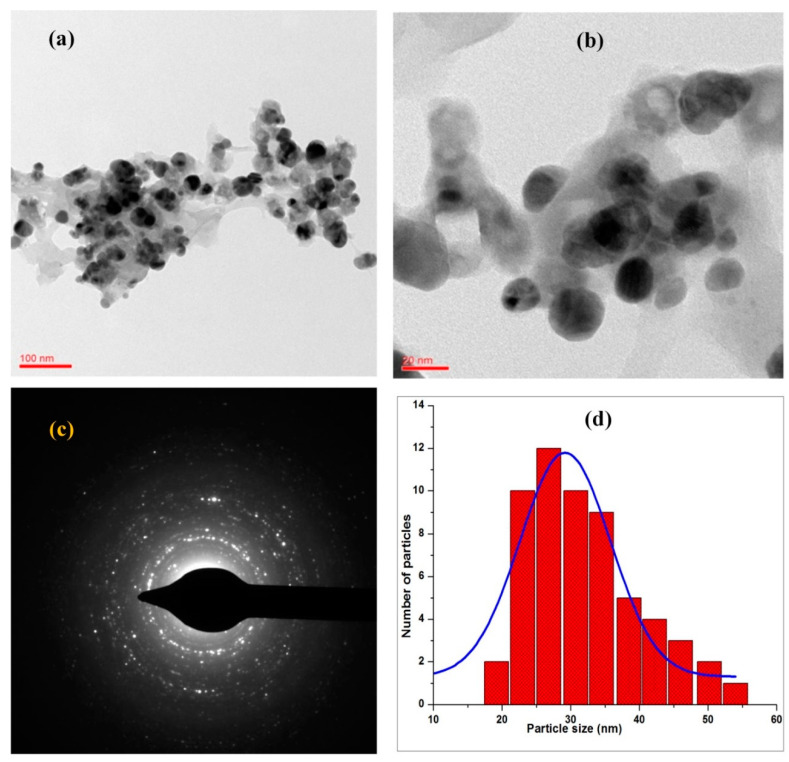
High-resolution transmission electron microscopy (HRTEM) images of GPE-AgNPs at (**a**) 100 and (**b**) 20 nm magnification, (**c**) selected area diffraction pattern (SAED), and (**d**) average particle size histogram of the synthesized GPE-AgNPs.

HR-TEM analysis was carried out to measure the particle diameter, size distribution, and crystallinity of biosynthesized GPE-AgNPs. The TEM micrographs of GPE-AgNPs at 100 and 20 nm magnifications are shown in Figure 3a,b. The synthesized GPE-AgNPs were found to be monodispersed, spherical, and uniformly distributed in the sample with no significant aggregation (Figure 3b). The separation between the AgNPs within the aggregates seen in the HR-TEM image could be due to good biomolecules capping effect of GPE, which indicates successful synthesis of GPE-AgNPs (Figure 3b). The selected area electron diffraction (SAED) with spots superimposed on rings revealed polycrystalline nature of AgNPs (Figure 3c). The SAED model confirmed the presence of elemental AgNPs and also agreed with the XRD analysis results. The maximum nanoparticles were in the size ranging between 22 and 35 nm by histogram (Figure 3d).

### 3.4. In Vitro Antioxidant Assays

Antioxidants are of incredible favorable position in improving the human health by averting or delaying the beginning of various diseases by lowering the oxidative stress, genetic mutations, and other influencing parameters related to cell damage. There is a huge interest increased in the application of antioxidants as dietary supplements from natural source instead of synthetic sources to protect from various degenerative diseases [41,42]. The present examination was to research the antioxidants action of GPE-AgNPs in terms of DPPH and ABTS radical scavenging activity. DPPH radical scavenging assay is a reliable procedure for the evaluation of antioxidant capacity of natural or synthetic compounds. DPPH is a constant free-radical eminent for stage function in diminution of accommodating hydrogen from donors [43].

**Figure 4:**
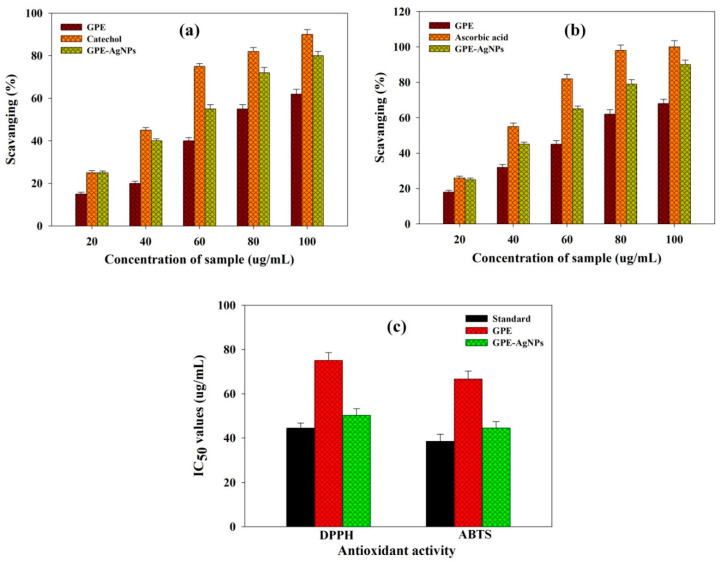
Percentage of (**a**) 2,2-diphenyl-1-picryhydrazyl (DPPH), (**b**) 2,2′-Azino-bis(3-ethylbenzothiazoline)-6 sulfonic acid (ABTS) radical scavenging potential by GPE, standard catechol, and biosynthesized GPE-AgNPs at various concentration, and their (**c**) IC_50_ values.

The DPPH scavenging activity by GPE-AgNPs was surveyed based on the change in color. The change in color might be due to the scavenging of DPPH owing to contribution of hydrogen or electron is accountable after addition of GPE-AgNPs into the solution of DPPH [44]. In this study, GPE and GPE-AgNPs at different concentrations range showed enhanced DPPH radical scavenging activity in a concentration reliant mode (Figure 4a). The radical scavenging activity of GPE-AgNPs was higher in comparison with GPE and comparable with standard catechol, and the IC_50_ values of 50.0 ± 2.25, 75.2 ± 3.15, and 44.4 ± 1.25 μg/mL, respectively, were found (Figure 4c).

In addition, the biosynthesized GPE-AgNPs were scrutinized for their antioxidative prospective according to the assay performed in ABTS free radical scavenging; the results are displayed in Figure 4b. The GPE-AgNPs showed effective scavenging of the ABTS radical in a concentration-dependent manner. The GPE-AgNPs and catechol IC_50_ values were found to be similar of about 38.46 ± 1.41 and 44.44 ± 2.35 μg/mL, respectively, whereas GPE was found less effective with higher IC50 value (66.66 ± 2.25 μg/mL). The significant DPPH and ABTS scavenging potential of GPE-AgNPs might be attributed to the presence of various phytoconstituents, specially TPC and TFC. These phytoconstituents may get adsorbed onto the active surfaces of AgNPs as capping agent, which increases the surface area, thereby interacts and scavenge these free radicals efficiently [45]. Khoshnamvand et al. [46] showed that ascorbic acid and polyphenolic compounds of *Allium ampeloprasum* leaves extract significantly enhanced the free radical scavenging activities by donating hydrogen atoms and electrons. 

### 3.5. In Vitro Antidiabetic Activity

**Figure 5:**
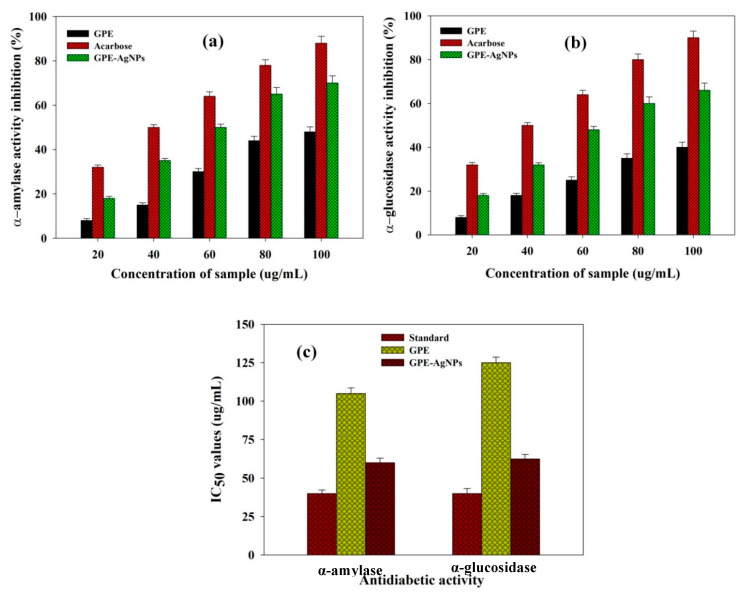
Inhibitory percentage of (**a**) α-amylase and (**b**) α-glucosidase enzyme activity by GPE, standard acarbose, and biosynthesized GPE-AgNPs at various concentration and their (**c**) IC50 values.

Diabetes is still life-threatening because of limited therapeutic efficacy of available synthetic drugs. The number of diabetic patients is anticipated to be 300 million worldwide by 2025 and thus, narrative approaches are requisite to prevent and to treat diabetes mellitus [47]. The starch-related enzymes in stomach, mainly, pancreatic α-amylase and intestinal α-glucosidase, have potential in the breakdown of polysaccharides and disaccharides into monosaccharides, and its absorption leads to increase in blood sugar level [48]. The restraint of these catalysts is particularly important and is effectual strategy for the handling of noninsulin-dependent type-2 diabetes since it hinders the influx of glucose in the blood [49]. The GPE-AgNPs at various concentrations potentially inhibited α-amylase and α-glucosidase enzymes activities in a dose reliant mode (Figure 5a,b). With increasing concentration of GPE-AgNPs from 20 to 100 µg/mL showed maximum enzymatic inhibition in both enzyme activities of about 70% and 65%, respectively, whereas GPE showed moderate inhibition (48% and 40%) in both enzyme activities. The IC_50_ values of GPE, standard acarbose, and synthesized GPE-AgNPs for α-amylase and α-glucosidase are shown in Figure 5c.

The synthesized GPE-AgNPs showed significant both α-amylase and α-glucosidase enzyme inhibitory activity. The IC_50_ values of GPE-AgNPs and acarbose was observed to be 60.2 and 40.2 μg/mL for α-amylase and 62.5 and 40.0 μg/mL for α-glucosidase, respectively. The synthesized GPE-AgNPs diminished the degrees of enzyme activities, which are in charge of catalyzing the hydrolysis of complex sugars and expanded the utilization pace of glucose stated by some investigators [28,50]. The foregoing results showed biosynthesized GPE-AgNPs exhibits significant inhibitory enzyme activity for both α-amylase and α-glucosidase may because of polyphenolic compounds of GP that are present in the AgNPs as capping agent which form complexes with the enzyme resulting in alteration of their biological structures and their catalytic activity [47]. It was also supposed that AgNPs have ability for protein corona formation which results in protein unfolding and their functions consequently leads to enzyme inhibitory performance by GPE-AgNPs [51]. The obtained results also exhibit the potential usefulness of biosynthesized GPE-AgNPs to treat diabetes mellitus and its associated complications. Utilization of GPE-AgNPs can be considered as safe, economical and an effective approach for diabetes care.

### 3.6. In Vitro Antibacterial Activity

Antimicrobial resistance is one of the most serious global public health threats in the present age, and there is an urgent need to extend novel antimicrobial methodologies to take care of multidrug-resistant microbial strains. Grape pomace extract and its respective synthesized GPE-AgNPs were tested against clinical bacterial pathogens. The antibacterial activity of biosynthesized AgNPs against the selected microbial strains was quantitatively determined on the basis of the ZOI (Table 2). Increasing the concentration of GPE-AgNPs showed sequential increase in ZOI for both the strains (Table 2). The inhibition of bacterial growth in the region of the disc was because of liberation of soluble inhibitory biomolecules from the phytofabricated AgNPs. The phytoconstituents in the biogenic AgNPs could play a vital role in showing the antibacterial activity of the AgNPs against the selected microbial strains. In our study, the synthesized GPE-AgNPs demonstrated maximum zone of inhibition aligned with Gram-negative (*E. coli*) relative to Gram-positive (*S. aureus*) microorganisms. The distinction in affectability of Gram-positive and Gram-negative microorganisms to GPE-AgNPs was because of the thickness and the constituents of their membrane structure [33]. The obtained results are found similar with the earlier studies concerning AgNPs synthesized using biological materials [9,52].

### 3.7. Synergistic Effect of GPE-AgNPs with Commercial Antibiotics

The potential synergistic effect of GPE-AgNPs with five commercial antibiotics (ampicillin, tetracycline, kanamycin, vancomycin, and streptomycin) was investigated against *E. coli* and *S. aureus* through disc diffusion method. Details of the enhanced activity in synergy with antibiotics and activity index of GPE-AgNPs against both the microbial strains are depicted in Table 3. GPE-AgNPs with ampicillin, tetracycline, and streptomycin exhibited increased synergistic antibacterial activity. In combination with vancomycin, moderate increase in antibacterial activity was recorded, whereas kanamycin showed null effect for both *E. coli* and *S. aureus* (Table 3). Similar kind of results was observed in other studies [26,53]. Such collegial effect of GPE-AgNPs in combination with commercial antibiotics would be a helpful approach to lessen the excessive use of antibiotics to control multidrug-resistant microbial species.

### 3.8. Mechanism of Antibacterial Activity by GPE-AgNPs

**Figure 6:**
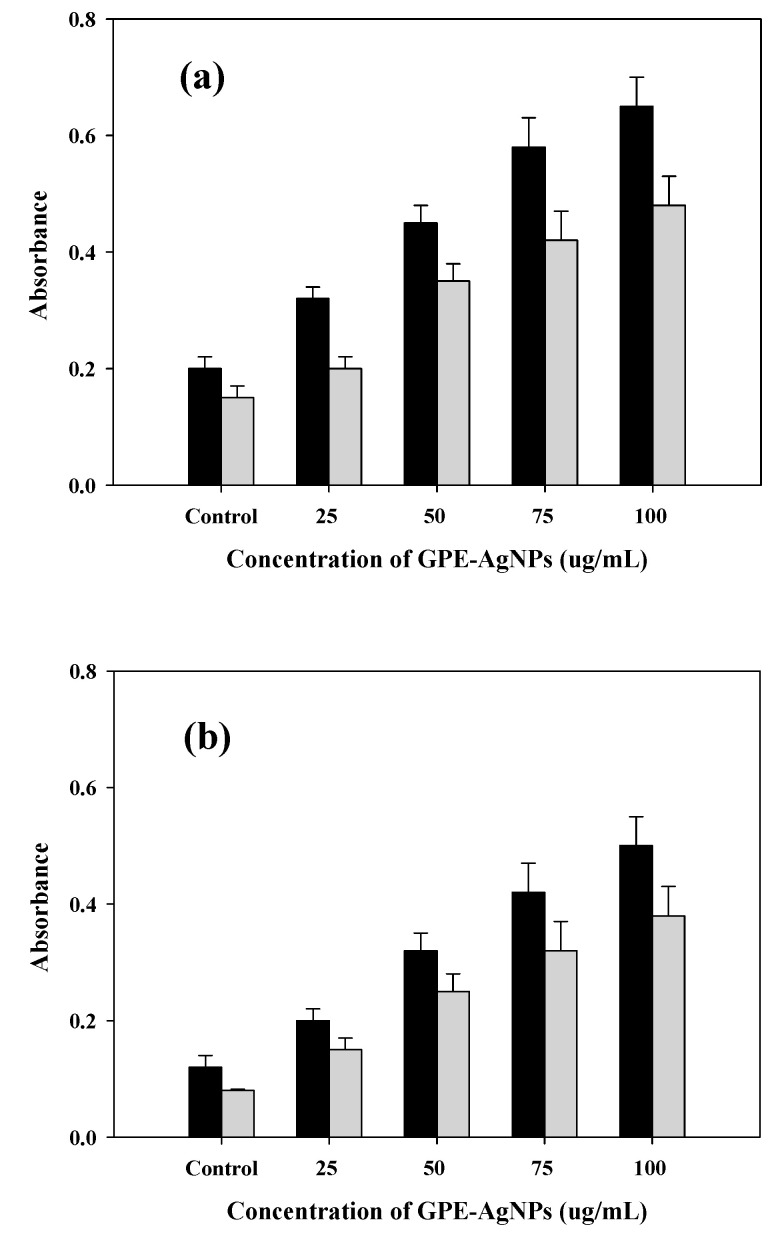
Release of absorbing materials from the cell suspensions at 260 nm (black bar) and 280 nm (gray bar) of (**a**) *E. coli* and (**b**) *S. aureus* bacteria treated with different concentration of GPE-AgNPs after 6 h of incubation.

Release of intracellular contents such as nucleic acid components, proteins, reducing sugars, etc. in the presence of NPs due to bacterial cell membrane integrity damage is a well-observed phenomenon [31]. The superior antibacterial potential of silver nanoparticles was due to the elevated surface area to volume ratio of NPs resulting in more surface exposure to the bacterial cell surface. Moreover, NPs can easily penetrate in the bacterial cell through the cell membrane and thereby the AgNPs interacts with the intracellular materials including genetic material and important biomolecules resulting in the cellular toxicity leading to cell death [32,54]. The discharge of intracellular materials that absorb at 260 nm which is a sign of membrane integrity damage [31,55]. Therefore, we have investigated the effect of GPE-AgNPs on the membrane integrity of *E. coli* and *S. aureus*. After exposure to GPE-AgNPs, the absorbance values at 260 and 280 nm were recorded to understand the release of cellular contents, which is also sign of damage of cell membrane. The results are presented in Figure 6a,b. With the increase in concentration of GPE-AgNPs, sequential increase in absorbance for both the strains was recorded (Figure 6a,b). Thus, cell membrane damage by GPE-AgNPs is concentration dependent. The results observed were in agreement with the findings to the previous studies [31,54,55].

**Figure 7:**
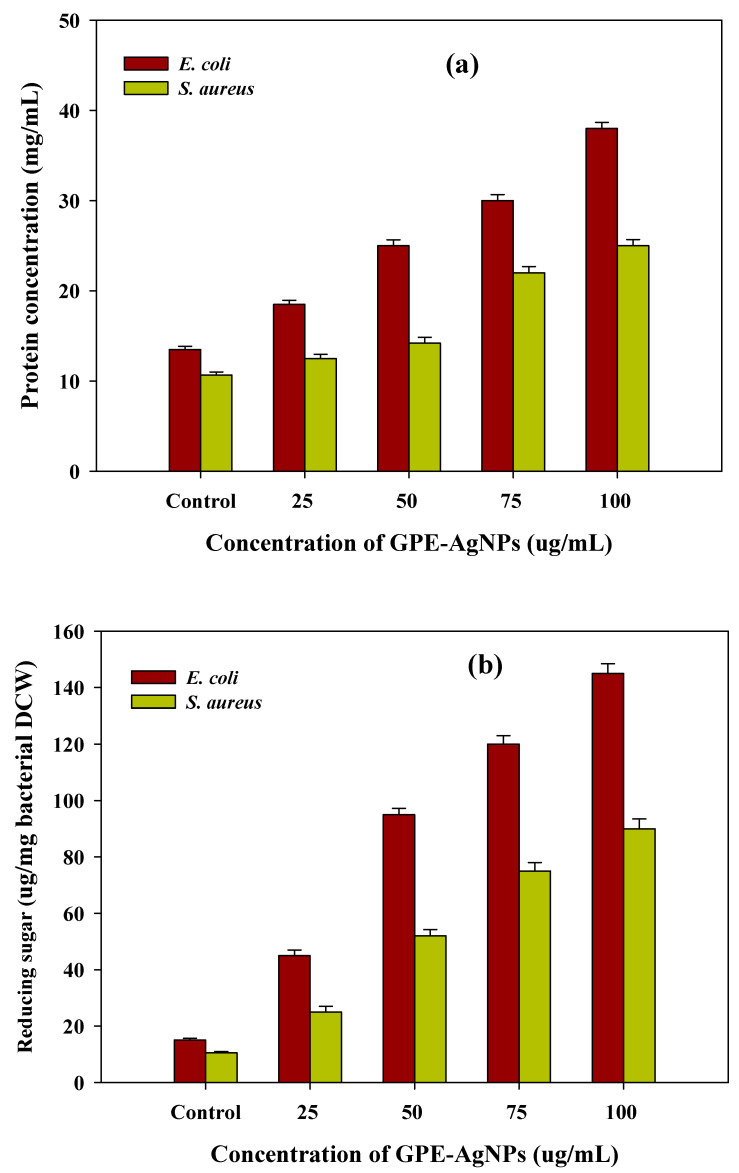
Cellular leakage analysis of (**a**) protein content and (**b**) reducing sugar from the cell suspensions of *E. coli* and *S. aureus* bacteria treated with different concentration of GPE-AgNPs after 6 h of incubation.

The mechanism lies behind antibacterial activity after GPE-AgNPs treatment was determined by measuring cellular leakages in terms of reducing sugars and proteins. After treatment of GPE-AgNPs at different concentration (25 to 100 μg/mL) in both bacteria, sequential increase in proteins and reducing sugars concentration was observed. The maximum values of proteins (38.0 and 25.0 mg/mL) and reducing sugar concentration (145 and 90 μg/mg of bacterial dry cell weight) at higher GPE-AgNPs concentration for *E. coli* and *S. aureus*, respectively, was observed (Figure 7a,b).

The obtained results were found to be significantly higher than untreated control cells (13.5 mg/mL and 10.65 μg/mg of bacterial dry cell weight) (Figure 7a,b). In addition, higher cellular leakage in *E. coli* as compared to *S. aureus* was recorded. Similar kind of result was found where higher antibacterial sensitivity in Gram-negative bacterial strains as compared to Gram-positive bacteria was seen, which supports our results [32,56,57]. In conclusion, the results suggest that GPE-AgNPs showed ability to penetrate and damage the bacterial cell membrane integrity, thus, disrupting the cytoplasmic biomolecules results in discharge of cytoplasmic elements, which are concentration dependent. Still more research work is needed to get the exact underlying mechanism of antibacterial activity by synthesized NPs.

## 4. Conclusions

The proposed work emphasizes on the efficient and greener fabrication of silver nanoparticles synthesis using fruit waste grape pomace. The biofabricated AgNPs possessed the added advantage of incorporation of active biomolecules as capping agent, which proves it as potent free radical scavengers and antidiabetic agent. Moreover, the synthesized GPE-AgNPs exhibited noteworthy antibacterial potential and synergistic activity in combination with standard antibiotics against human infectious bacterial pathogens. The details of antibacterial mechanism were investigated by measuring the membrane integrity and cellular biomolecules leakage. The outcome of the research confirms the low-cost fabrication process using waste GP, making it more cost-effective and eco-friendly method, and also offers alternative therapeutic preferences with many advantages such as safe biomedical application with no side effects.

## Figures and Tables

**Table 1:** Total polyphenols and total flavonoids contents in grape pomace solvent extract.

Name of Phytochemicals	Concentration
Total polyphenol (TPC)	32.38 ± 2.85 (mg of GAE/g of GPE)
Total flavonoids (TFC)	14.65 ± 0.85 (mg of QE/g of GPE)

Values are means of three experiments ± SE.

**Table 2:** In vitro antibacterial activity of grape pomace extract (GPE) and synthesized GPE-silver nanoparticles (AgNPs) against *Escherichia coli* and *Staphylococcus aureus*.

GPE-AgNPs Concentration (μg/mL)	Zone of Inhibition (mm)	
	*Escherichia coli*	*Staphylococcus aureus*
GPE extract (20)	5.25 ± 0.45	3.55 ± 0.18
10	8.85 ± 0.75	6.42 ± 0.54
20	12.8 ± 0.65	10.4 ± 0.72
30	18.5 ± 0.82	14.2 ± 0.92

Values are mean ± standard error of three replicates.

**Table 3:** Enhanced antibacterial activity of synthesized GPE-AgNPs in combination with standard antibiotics against *Escherichia coli* and *Staphylococcus aureus*.

	Zone of Inhibition (mm)			
Name of Commercial Antibiotics	*Escherichia coli*		*Staphylococcus aureus*	
	Antibiotics	Antibiotics + GPE-AgNPs	Antibiotics	Antibiotics + GPE-AgNPs
Ampicillin	12.5 ± 0.68	18.5 ± 0.85	10.2 ± 0.45	15.4 ± 0.68
Tetracycline	14.5 ± 0.65	20.2 ± 0.88	10.5 ± 0.55	15.6 ± 0.72
Kanamycin	10.5 ± 0.72	12.8 ± 0.65	8.50 ± 0.41	11.1 ± 0.40
Vancomycin	11.2 ± 0.77	14.5 ± 0.71	9.22 ± 0.39	11.5 ± 0.42
Streptomycin	13.2 ± 0.82	17.8 ± 0.74	11.2 ± 0.44	14.8 ± 0.62

Values are mean ± standard error of three replicates.
